# Medical Items

**Published:** 1913-01

**Authors:** 


### MEDICAL ITEMS

### THE RELATION OF IRON TO ANEMIA IN INFANCY AND CHILDHOOD

Ashby has this to say in the Lancet for July 20, 1912, as to the absorption of iron:

The body gets the iron necessary for its needs through the food; the different foods, however, vary a good deal in the amount of iron which they contain. Thus, comparing the amount of iron in different foods: beef, 0.02; wheat, 0.026; potato, 0.042; peas, 0.024; yolk of eggs, 0.04; milk, human and cow, 0.003 per

